# Associations of meal timing and sleep timing with premenstrual syndrome and dysmenorrhea in Japanese women: a cross-sectional study

**DOI:** 10.3389/fnut.2026.1875559

**Published:** 2026-07-24

**Authors:** Aleef Daniel, Yu Nishimura, Yun-Peng Lo, Ariko Umezawa, Noriko Sato, Akiko Furutani, Eiichi Yoshimura, Mikiko Michie, Shigenobu Shibata, Tatsuhiko Kubo, Yu Tahara

**Affiliations:** 1 Department of Public Health and Health Policy, Graduate School of Biomedical and Health Sciences, Hiroshima University, Hiroshima, Japan; 2 Department of Nutritional Science, Faculty of Food and Nutritional Sciences, Japan Women's University, Tokyo, Japan; 3 Faculty of Home Economics, Aikoku Gakuen Junior College, Tokyo, Japan; 4 Laboratory of Nutrition Metabolism, Research Center for Clinical Nutrition, National Institutes of Biomedical Innovation, Health and Nutrition, Osaka, Japan; 5 Asken Inc., Shinjuku, Tokyo, Japan; 6 School of Advanced Science and Engineering, Waseda University, Tokyo, Japan

**Keywords:** chrono-nutrition, chronotype, circadian rhythms, dysmenorrhea, meal timing, menstrual pain, premenstrual syndrome, sleep timing

## Abstract

**Background:**

Premenstrual syndrome (PMS) and dysmenorrhea (menstrual pain) are common among women of reproductive age. Fluctuations in reproductive hormone levels during the late luteal phase have been linked to changes in eating and sleep patterns, which may contribute to PMS and dysmenorrhea. This study examined the associations of meal timing and sleep timing with PMS symptoms and dysmenorrhea.

**Methods:**

In this cross-sectional study, 426 Japanese women (mean age: 33 ± 5 years; body mass index: 22.2 ± 4.3 kg/m^2^) were recruited via the Asken mobile health application. Participants completed questionnaires assessing PMS symptoms, dysmenorrhea, meal timing (time of first meal, time of last meal, and eating window), sleep timing (wake-up time, bedtime, and sleep duration), chronotype (individual preference for earlier or later sleep-wake timing), and social jetlag (differences between sleep timing on workdays and free days). Multivariable regression models were used after adjusting for age, BMI, smoking status, and alcohol consumption.

**Results:**

On workdays, later first meal (β = 0.211) and earlier wake-up time (β = −0.184) were associated with higher eating-related PMS symptoms. On free days, earlier first meal (β = −0.191), later last meal (β = 0.162), longer eating window (β = 0.133), later wake-up time (β = 0.308), and longer sleep duration (β = 0.147) were associated with higher dysmenorrhea severity. Lower breakfast frequency (β = −0.13) was associated with higher eating-related PMS symptoms. Higher afternoon snacking was associated with higher total PMS symptoms (β = 0.13), higher eating-related PMS symptoms (β = 0.11), and higher sleep-related PMS symptoms (β = 0.11). More frequent nighttime snacking was associated with higher odds of moderate-to-severe PMS symptoms (OR = 1.11, 95% CI = 1.00–1.23).

**Conclusions:**

Meal and sleep timings were differentially associated with PMS symptoms and dysmenorrhea across workdays and free days. Workday behaviors were primarily associated with eating-related PMS symptoms, whereas free-day behaviors were primarily associated with dysmenorrhea severity. These findings suggest that menstrual symptoms may be differentially associated with behaviors shaped by socially imposed schedules and circadian preferences. Future longitudinal and interventional studies are needed to clarify causal relationships between meal timing, sleep timing, and menstrual health.

## Graphical Abstract

**Figure:**
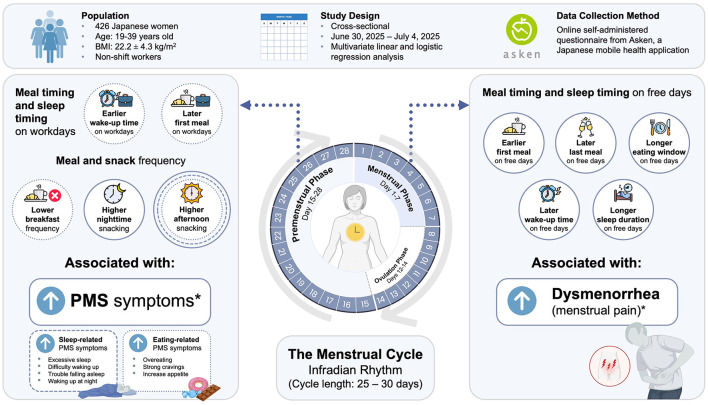
Graphical summary of associations between meal timing, sleep timing, with PMS symptoms and dysmenorrhea. Earlier wake-up times and later first meal on workdays, as well as lower breakfast frequency, and increased afternoon and nighttime snacking were associated with higher PMS symptoms, particularly eating-related PMS symptoms. On free days, earlier first meal, later last meal, longer eating windows, later wake-up times, and longer sleep duration were associated with greater dysmenorrhea severity. The central panel depicts the menstrual cycle as an infradian rhythm, with cycle lengths between 25 to 30 days. Similar line styles indicate statistically significant associations. Created with BioRender.com.

## 1 Introduction

Menstrual health is a fundamental component of women's overall health. However, it remains one of the most substantially understudied areas in biomedical research (1, 2). This gap reflects not a lack of clinical relevance, but a longstanding underrepresentation of menstrual health and women of reproductive age across all areas of health research (3). Premenstrual syndrome (PMS) and dysmenorrhea (menstrual pain) are two of the most prevalent and burdensome, yet insufficiently understood, menstrual health conditions in women of reproductive age.

About 95% of Japanese women suffer from PMS, with the prevalence of moderate-to-severe PMS and premenstrual dysphoric disorder (PMDD) to be 5.3% and 1.2%, respectively (4, 5). PMS comprises psychological and physical symptoms that emerge during the late luteal phase or premenstrual phase of the menstrual cycle. PMS typically occurs 1 to 2 weeks before menstruation, resolves within a few days after onset, and are absent in the week following menstruation (6, 7). These symptoms include anxiety, depression, impaired concentration, reduced energy, sleep disturbances, appetite changes, overeating, headaches, and somatic pain (8). Dysmenorrhea is characterized by cyclic pelvic pain associated with menstruation (9). Globally, the pooled prevalence of dysmenorrhea has been estimated at approximately 70%: similar rates have been observed across countries regardless of economic status (9, 10). Both PMS and dysmenorrhea are associated with reduced quality of life, impaired academic and work performance, and increased healthcare utilization, representing as substantial but often overlooked public health burden (10).

The menstrual cycle represents an infradian rhythm, which is defined as a biological rhythm with a period longer than 24 h, with an average duration of 28 days and typical cycle lengths ranging between 25 and 30 days (11, 12). This infradian rhythm of the menstrual cycle interacts with the circadian rhythm, which is the 24-h rhythm of physiology (13, 14). Almost all living organisms have adapted to the Earth's 24-h day and night cycle by developing an internal biological clock known as the circadian rhythm (15). Most of the behaviors and biological processes essential for healthy physiological functioning are synchronized with the circadian rhythm. This synchronization involves 1) daytime activity and eating and 2) nighttime sleep and fasting (16). However, industrialization has disrupted these behavioral patterns. Widespread access to artificial lighting has led to increased wakefulness and reduced nighttime sleep. Advanced food production and preservation have made food available around the clock (17). These changes in eating and sleep behavior led to circadian misalignment. The circadian system plays a central role in hormonal secretion, including estrogen (18), inflammatory response (19), pain perception (20), and mood regulation (21), all of which are implicated in PMS and dysmenorrhea pathophysiology.

Circadian rhythms may be particularly vulnerable to disruption during the late luteal phase due to the decline in the estrogen and progesterone levels (6). Fluctuations in female reproductive hormones across the menstrual cycle are recognized as key factors to sex differences in both mood and sleep disorders (22). Women exhibit approximately twice the lifetime risk of developing mood disorders such as depression and anxiety, and a 1.25-fold greater overall risk of sleep disruption compared to men (7). Estrogen is associated with more stable circadian rhythm and high levels of progesterone reduce anxiety and promote sleep (6, 8). These hormonal changes during the luteal phase have been linked with poor sleep and impaired mental health and pain sensitivity (23–25). Dysmenorrhea may further exacerbate sleep disruption and mood disorders (26), suggesting a bidirectional relationship between menstrual symptoms and circadian rhythms. Together, these findings highlight the importance of identifying modifiable behavioral factors, such as sleep and mental health, in managing menstrual symptoms.

Another potentially modifiable behavioral factor is the timing of food intake. Chrono-nutrition has emerged as an important field for examining how the alignment of food intake timing with circadian rhythms influences health outcomes. While the central circadian clock is primarily synchronized by light exposure, feeding-fasting cycles act as important zeitgebers for peripheral metabolic clocks; therefore, disruption of these can lead to circadian misalignment (27). Modern lifestyles characterized by irregular eating patterns and disrupted sleep–wake cycles may further impair circadian regulation and promote misalignment between the internal circadian rhythm and the external light-dark cycle (28, 29). Previous studies have suggested that eating habits and sleep behavior, such as skipping breakfast, eating disturbances, poor sleep quality, and daytime sleepiness, may influence menstrual disorders and PMS symptoms (30–33). The Premenstrual Syndrome Questionnaire (PSQ), developed and validated for the Japanese population by Takeda et al. (5), includes PMS symptoms related to appetite and sleep disturbances. Therefore, in addition to total PMS symptom severity, the present study examines eating-related symptoms (increased appetite, food cravings, and overeating) and sleep-related PMS symptoms (excessive sleepiness, difficulty waking, and poor sleep quality) as separate symptom domains.

Most previous studies examining sleep and nutrition in relation to menstrual symptoms have not incorporated the timing of eating and sleep behaviors. Chronotype and social jetlag may provide additional insights into the influence of circadian alignment on menstrual health. Chronotype reflects individual preferences for earlier or later sleep-wake timing, while social jetlag is the differences between sleep timing on workdays and free days reflecting variability in daily schedules (34, 35). Late chronotype and higher social jetlag have been reported to be associated with more severe menstrual symptoms as well as adverse cardiometabolic, psychological, and sleep-related outcomes (36, 37). However, to date, no study has comprehensively evaluated the combined roles of meal and snack timing, sleep timing, chronotype, and social jetlag in relation to both PMS symptoms severity and dysmenorrhea. This has resulted in a critical gap in understanding how these modifiable behavioral timing factors shape menstrual health.

To address this gap, we conducted a cross-sectional study examining meal timing, sleep timing, chronotype, and social jetlag and their associations with PMS symptoms and dysmenorrhea among women of reproductive age.

## 2 Material and methods

### 2.1 Study design

This cross-sectional study used data from a widely used Japanese food-logging and health mobile application, Asken, in Japan, which had over 12 million user registrations as of January 2026. Asken has been used in numerous peer-reviewed studies and has demonstrated acceptable reliability for self-reported food logs in assessing lifestyle behaviors, including dietary habits (38). The study was approved by the Ethics Review Committee on Research with Human Subjects at Hiroshima University (E2024-0254) and was conducted in accordance with the Declaration of Helsinki. All participants provided informed consent electronically prior to participation.

### 2.2 Participants recruitment and demographic variables

Women aged 19–39 years were recruited between June 30 and July 4, 2025, using the Asken mobile application. The eligibility criteria included reproductive age and current residence in Japan. In total, 538 participants completed the survey. After excluding shift workers (*n* = 44), and those with missing data (*n* = 68), 426 participants were included in the final analysis. Shift workers were excluded because of their irregular work schedules, which are associated with circadian misalignment and may confound the associations between meal timing and sleep timing with menstrual outcomes (39–41). Data on age, height, weight, shiftwork status, and lifestyle factors, such as smoking and alcohol consumption, were collected through an online self-administered questionnaire.

### 2.3 Sample size calculation

The required sample size was estimated to be 139 participants using G^*^Power for linear multiple regression analysis, assuming a medium effect size (*f*^2^ = 0.15), a significance level (α) of 0.05, 10 predictors, and a statistical power (1–β) of 0.80. This estimation was based on previous studies reporting associations between sleep behavior and PMS symptoms (32, 42).

### 2.4 PMS symptoms and dysmenorrhea

PMS symptoms were assessed using the Premenstrual Symptoms Questionnaire (PSQ) validated for the Japanese population (5). Participants were asked to report symptoms experienced during 1–2 weeks before menstruation, which is the luteal phase. Participants rated the severity of psychological and physical symptoms (items 1–11) and three functional impairment items (items 12–14) occurring during the luteal phase on a 4-point scale (none, mild, moderate, and severe). The total PMS score was calculated according to established scoring criteria, ranging from 11 to 56. For regression analyses, the total PMS score was treated as a continuous variable. Moderate-to-severe PMS status was defined according to the PSQ criteria. Moderate-to-severe PMS was identified when all of the following conditions were met: (1) at least one of the core emotional symptoms (items 1–4) was rated as moderate or severe; (2) at least five of the 11 symptoms (items 1–11) were rated as moderate or severe; (3) at least one functional impairment item (items B1-B3) was rated as moderate or severe; and (4) symptoms improved within a few days after the onset of menstruation (5). In addition to the total PMS severity score and moderate-to-severe PMS status classification, individual PSQ symptom items and functional impairment items were analyzed descriptively (Supplementary File 1). Given that the PSQ encompasses multiple symptom domains, including eating- and sleep-related symptoms, we specifically examined the associations between meal timing and sleep timing and the eating- and sleep-related PSQ subdomains (item 10: eating-related; item 9: sleep-related). These subdomains were selected based on *a priori* selection based on their relevance and direct relationship to the primary exposures of interest, such as meal timing and sleep timing. Dysmenorrhea severity was assessed using a 4-point verbal rating scale (VRS): 1 = none; 2 = slight interference with work or academic activities (mild); 3 = interference with work or academic activities to the extent that you want to lie down and rest temporarily (moderate), and 4 = unable to work or attend school (severe), as developed in previous studies (43). This scale is an ordinal grading system originally adapted from Biberglou et al., and Andersch et al., and has been widely used in gynecological studies to classify dysmenorrhea severity (44–46). Participants rated the intensity of menstrual pain during their most recent menstrual cycle. For regression analysis, the severity of dysmenorrhea was treated as a continuous variable as an approximation, acknowledging that the scale is ordinal.

### 2.5 Meal timing

Participants reported habitual meal timing that include the time of the first meal, the time of the last meal, and the eating window (47). Eating window duration was calculated as the time difference from the start of the first eating event to the end of the last eating event. The first meal is defined as the first eating event of the day, regardless of whether it was breakfast, lunch, dinner or another meal (48). All meal timing variables were calculated separately for workdays and free days. If the time of the last meal was earlier than the time of the first meal, 12 h were added to keep the timing within the same day and to avoid negative eating window values.

### 2.6 Sleep timing, chronotype (MSFsc), and social jetlag

Sleep timing was assessed using the short version of the Munich Chronotype Questionnaire (34). Participants reported habitual sleep onset and wake-up times separately for workdays and free days. Sleep duration was estimated as interval between self-reported onset and wake-up time and therefore reflects estimated sleep duration rather than time spent in bed.

From these data, the mean sleep duration, sleep duration on free days, midpoint of sleep on free days (MSF), and sleep-corrected midpoint of sleep on free days (MSFsc) were calculated according to established procedures. MSFsc was used as an indicator of the chronotype, with later values reflecting a later circadian preference. Social jetlag was calculated as the absolute difference between the midpoints of sleep on workdays and free days. Larger values indicated a greater misalignment between endogenous circadian preference and socially imposed schedules (34).

### 2.7 Covariates

Multivariate models were adjusted for potential confounders selected *a priori* based on biological plausibility and prior literature. The covariates included age, BMI, smoking status (current smoker vs. non-smoker), and alcohol consumption (yes/no). These covariates are lifestyle factors known to be associated with eating and sleep behavior, as well as menstrual symptoms (30–33).

### 2.8 Statistical analysis

All statistical analyses were performed using R software and GraphPad Prism 9.0. Statistical significance was defined as a two-sided *p*-value < 0.05. Statistical significance is indicated by ^*^p < 0.05, ^**^p < 0.01, ^***^p < 0.001, and ^****^p < 0.0001. Continuous variables were assessed for normality using the Shapiro–Wilk test. Normally distributed variables are presented as mean ± standard deviation (SD), and non-normally distributed variables are presented as median (interquartile range, IQR). Categorical variables were presented as frequencies and percentages. Group comparisons were conducted using independent *t*-tests or one-way analysis of variance (ANOVA) for normally distributed variables, and Wilcoxon rank-sum or Kruskal–Wallis tests for non-normally distributed variables.

Associations of meal timing, sleep timing, chronotype, and social jetlag with PMS symptoms and dysmenorrhea were examined using multivariate regression models. Multivariate linear regression was used for continuous outcomes, including the total PMS symptom severity. Dysmenorrhea severity, assessed using a 4-point ordinal scale, was treated as continuous variable for regression analysis, consistent with previous epidemiological studies. Standardized beta coefficients (β) and 95% confidence intervals (CIs) were reported. Multivariate logistic regression was used to examine associations with moderate-to-severe PMS status, and odds ratios (ORs) with 95% CIs were reported. All models were adjusted for age, BMI, smoking status, and alcohol consumption. Forest plots were generated to visualize standardized β coefficients and ORs with corresponding 95% CIs. Multicollinearity among independent variables was assessed using the variance inflation factor (VIFs). VIF values below five were indicative of absence of problematic multicollinearity.

## 3 Results

### 3.1 Demographic characteristics, total PMS score, eating-related PMS symptoms, sleep-related PMS symptoms, and dysmenorrhea score

Table 1 summarizes the characteristics of the 426 participants included in the study. Overall, participants were adult Japanese women with a mean age of 33 ± 5 years and a mean BMI of 22.2 ± 4.3 kg/m^2^. Most participants were non-smokers and reported infrequent alcohol consumption (less than once per month or none).

**Table 1:** Characteristics of participants.

Characteristic	Total (*n* = 426)
Age, years	33 ± 5
Body mass index (BMI), kg/m^2^	22.2 ± 4.3
Alcohol consumption	
Everyday	15 (3.2)
3–4 times/week	8 (1.7)
1–2 times/week	31 (6.6)
1–2 times/month	75 (16)
Once per month	136 (29)
No	205 (44)
Smoking status	
Quit	28 (6.0)
No	419 (89)
Yes	3 (4.9)
Circadian variables	
Chronotype (MSFsc), hh:mm	04:04 ± 1:43
Social Jetlag, hh:mm	01:17 ± 1:02
≥ 1 h	184 (43.2%)
< 1 h	242 (56.8%)
Menstrual symptom variables	
Premenstrual syndrome (PMS) score	28.56 [9.79]
with PMS	89 (20.9%)
No PMS	337 (79.1%)
Eating-related PMS (moderate-severe severity) frequency	227 (53.3%)
Sleep-related PMS (moderate-severe severity) frequency	92 (21.6%)
Dysmenorrhea score	1.9 ± 0.72
1 (none)	136 (29%)
2 (mild)	245 (52.2%)
3 (moderate)	80 (17.1%)
4 (severe)	8 (1.7%)

Data are presented as mean ± SD, median [IQR], or number (percentage). MSFsc, Mid-sleep on free days corrected for sleep debt. Social jetlag, difference between mid-sleep on workdays and free days.

Alcohol consumption varied, with nearly half of the participants reporting no alcohol intake (44%), followed by consumption once per month (29%), 1–2 times per day (16%), 1–2 times per week (7%), 3–4 times per week (2%), and daily consumption (3%). Most participants were non-smokers (89%). The mean chronotype (MSFsc) was 04:04 ± 1:43 h, and the mean social jetlag was 1:17 ± 1:02 h. A social jetlag of more than 1 h was reported by 43.2% (*n* = 184), and 56.8% (*n* = 242) reported a social jetlag of 1 h or less.

The median total PMS score was 28.56 [IQR 9.79]. When ranked according to the proportion reporting moderate-to-severe severity (combined moderate and severe), eating-related symptoms were most prevalent in this cohort: 53.3% of participants reported moderate-to-severe eating-related PMS symptoms, characterized by overeating, strong craving, and increased appetite. Meanwhile, 21.6% reported severe moderate-to-severe sleep-related PMS symptoms, characterized by excessive sleep, trouble waking up in the morning, difficulty falling asleep, and waking up at night (Table 1; Supplementary File 1).

More than half (52.2%) reported mild dysmenorrhea, which is having slight interferences with work or academic activities (Table 1). 17.1% reported pain severe enough to require temporary rest, and 1.7% reported being unable to work or attend school. Overall, the mean dysmenorrhea score was 1.9 ± 0.72. For pain management, 35.4% of participants reported not using analgesics or painkillers during menstruation, while the substantial proportion (41.6%) reported some level of use, about 1–2 times per menstrual phase (Supplementary File 1).

Appetite changes before menstruation were common, with 72.5% of participants reporting increased appetite, compared with 21.3% reporting no change and 1.3% reporting a decrease in appetite. For physical and health conditions, 46.2% of participants reported cold intolerance, 40.4% reported body weight changes of ≥ 3 kg within the past year, 27.4% reported a history of anemia, and 24.5% reported premenstrual weight gain of ≥ 2kg. A total of 18.7% reported none of these conditions. As multiple responses were allowed, percentages do not sum to 100% (Supplementary File 1).

### 3.2 Meal and sleep timing on workdays and free days

Table 2 illustrates that meal timing and sleep timing variables that are stratified by workdays and free days were significantly different. This distinction was made to account for behavioral differences driven by socially imposed schedules on workdays compared with the endogenous circadian preferences observed on free days. Participants consumed their first meal later on free days (median [IQR]: 09:30 [3.00]) than on workdays (median [IQR]: 07:30 [2.30]), *p* < 0.001. Although the median time of last meal was similar on workdays and free days, the distributions were significantly different (median [IQR]: 20:00 [2:00]), *p* < 0.013. Finally, the eating window was shorter on free days (median [IQR]: 10:30 [3.00]) than on workdays (median [IQR]: 12:15 [3.00] h), *p* < 0.001.

**Table 2:** Meal and sleep timing variables on workdays and free days.

Variable	Workdays	Free days	*p*-value
Meal timing	
First meal, hh:mm	07:30 [2:30]	09:30 [3:00]	< 0.001^***^
Last meal, hh:mm	20:00 [2:00]	20:00 [2:00]	0.013^*^
Eating window, hh:mm	12:15 [3.00]	10:30 [3:00]	< 0.001^***^
Sleep timing	
Wake-up time, hh:mm	06:30 [1:30]	08:30 [3:00]	< 0.001^***^
Bedtime, hh:mm	00:00 [2:00]	00:00 [2:30]	< 0.001^***^
Sleep duration, hh:mm	07:00 [1:30]	08:00 [2:00]	< 0.001^***^

Values are presented as median [IQR]. *P* values were obtained using the Wilcoxon signed-rank test. Asterisks denote statistical significance (^*^p < 0.05, ^***^p < 0.001).

Participants wake up later on free days (median [IQR]: 08:30 [3.00]) than on workdays (median [IQR]: 06:30 [1.30], *p* < 0.001). Although the median of bedtime was similar between workdays and free days, the distributions were significantly different (median [IQR]: 00:00 [2.00] vs. 00:00 [2:30]) *p* < 0.001). Lastly, the sleep duration was longer on free days (median [IQR]: 8:00 [2.00]) than on workdays (median [IQR]: 07:00 [1.30] h), *p* < 0.001.

### 3.3 Associations of meal and sleep timing with total PMS score, PMS eating-related symptoms, PMS sleep-related symptoms, and dysmenorrhea

**Figure 1:**
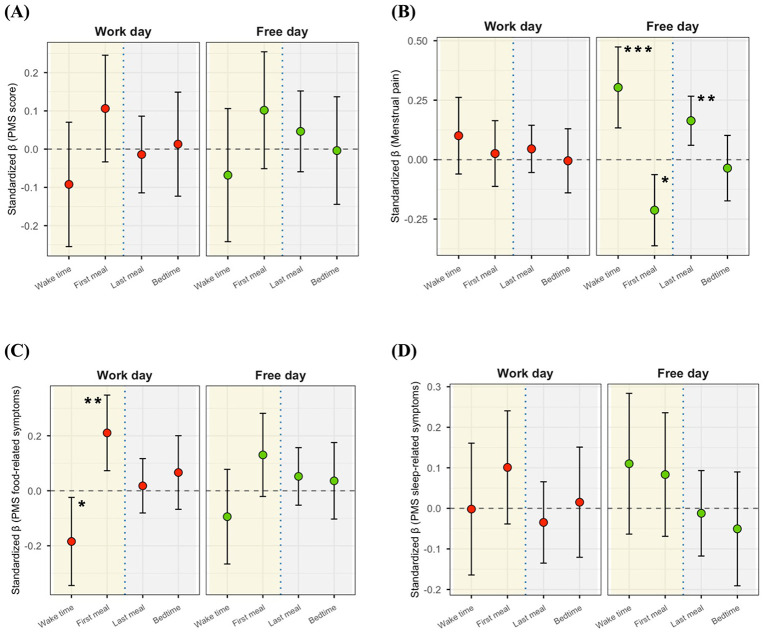
Associations of meal and sleep timing **(A)** total PMS score, **(B)** menstrual pain (dysmenorrhea), **(C)**, PMS eating-related symptoms (increased appetite, overeating, strong craving), and **(D)** PMS sleep-related symptoms (excessive sleep, difficulty waking up, and poor sleep quality). Values represent standardized regression coefficients (β) and 95% CI from multivariable linear regression models adjusted for age, BMI, smoking status, and alcohol consumption. Positive β values indicate higher symptom severity, whereas negative β values indicate lower symptom severity. The dashed horizontal line represents the null association (β = 0). Statistical significance is denoted by *p < 0.05, **p < 0.01, ***p < 0.001.

Linear regression analyses were conducted to examine the associations of meal timing and sleep timing with PMS symptoms and dysmenorrhea on workdays and free days after adjusting for age, BMI, smoking status, and alcohol consumption (Figure 1).

For the total PMS symptom score, no associations were observed between meal or sleep timing and PMS symptom score on either workdays or free days (Figure 1A). The models were not statistically significant for workdays (*R*^2^ = 0.013, adjusted *R*^2^ = −0.006, *p* = 0.691) or free days (*R*^2^ = 0.019, adjusted *R*^2^ ≈ 0, *p* = 0.445).

For dysmenorrhea, no associations were observed with meal or sleep timing and dysmenorrhea severity on workdays (Figure 1B). On free days, the overall model was statistically significant (*R*^2^ = 0.086, adjusted *R*^2^ = 0.059, *p* < 0.001). Later wake-up time (β = 0.308, *p* < 0.001) and later time of last meal (β = 0.162, *p* = 0.002) were positively associated with dysmenorrhea, whereas later time of first meal was inversely associated with dysmenorrhea (β = −0.191, *p* = 0.012). Bedtime was not associated with dysmenorrhea severity.

For eating-related PMS symptoms, earlier wake-up time (β = −0.184, *p* = 0.024) and later time of first meal (β = 0.211, *p* = 0.003) on workdays were associated with higher eating-related PMS symptoms (Figure 1C). No associations were observed for the time of last meal or bedtime. On free days, none of the meal or sleep timing variables was associated with eating-related PMS symptoms.

For sleep-related PMS symptoms, no associations were observed between meal and sleep timing variables on either workdays or free days (Figure 1D). The models were not statistically significant for workdays (*R*^2^ = 0.017, adjusted *R*^2^ = −0.001, *p* = 0.488) or free days (*R*^2^ = 0.033, adjusted *R*^2^ = 0.014, *p* = 0.086).

### 3.4 Associations of meal and sleep interval, eating window, and sleep duration with total PMS score, PMS eating-related symptoms, PMS sleep-related symptoms, and dysmenorrhea

**Figure 2:**
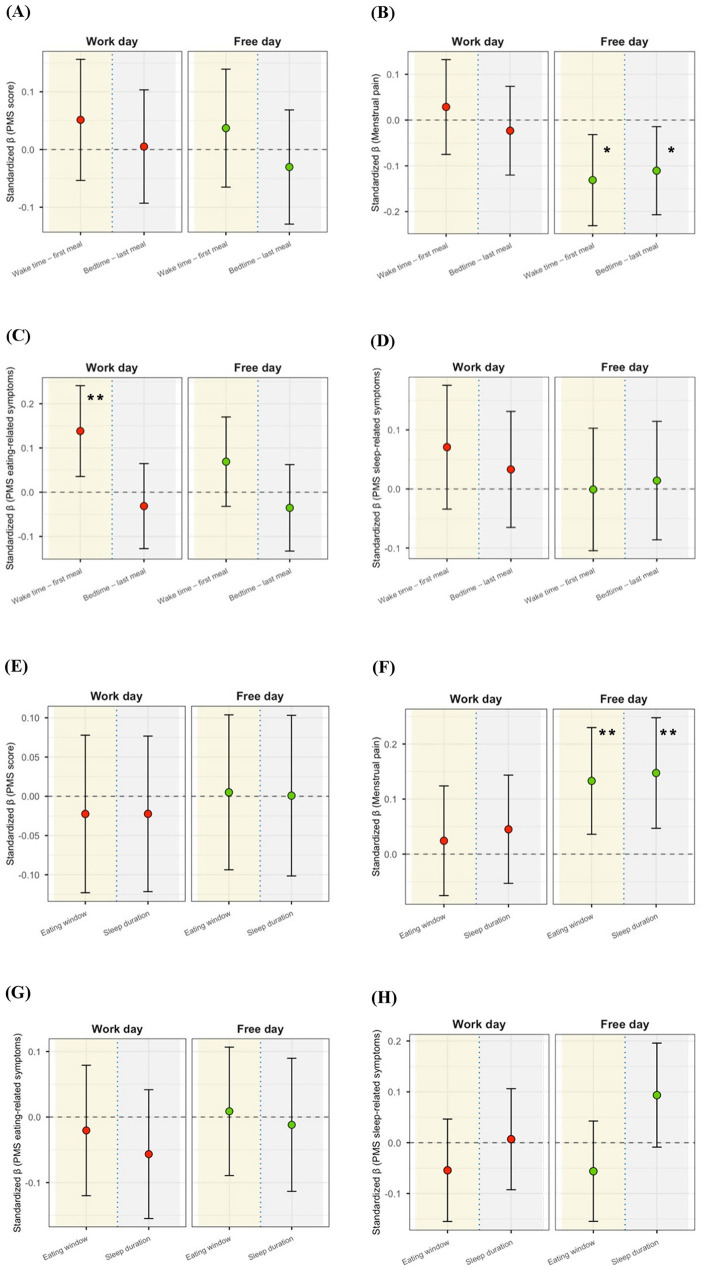
Associations of meal and sleep interval with **(A)** total PMS score, **(B)** menstrual pain (dysmenorrhea), **(C)** PMS eating-related symptoms (increased appetite, overeating, strong craving), and **(D)** PMS sleep-related symptoms (excessive sleep, difficulty waking up, and poor sleep quality). Associations of eating window and sleep duration with **(E)** total PMS score, **(F)** menstrual pain (dysmenorrhea), **(G)** PMS eating-related symptoms (increased appetite, overeating, strong craving), and **(H)** PMS sleep-related symptoms (excessive sleep, difficulty waking up, and poor sleep quality). Values represent standardized regression coefficients (β) and 95% CI from multivariable linear regression models adjusted for age BMI, smoking status, and alcohol consumption. Positive β values indicate higher symptom severity, whereas negative β values indicate lower symptom severity. The dashed horizontal line represents the null association (β = 0). Statistical significance is denoted by *p < 0.05, **p < 0.01, ***p < 0.001.

Linear regression analyses were conducted to examine the associations of meal and sleep interval timing with PMS symptoms and dysmenorrhea, after adjusting for age, BMI, smoking status, and alcohol consumption (Figure 2).

No clear associations were observed between meal and sleep interval timings and PMS scores on either workdays or free days (Figure 2A). The overall models were not statistically significant on workdays (*R*^2^ = 0.028, adjusted *R*^2^ = 0.002, *p* = 0.393) or free days (*R*^2^ = 0.032, adjusted *R*^2^ = 0.005, *p* = 0.290).

For dysmenorrhea, no associations were observed between meal and sleep interval timing, and dysmenorrhea severity on workdays (Figure 2B). On free days, longer wake–first meal intervals and longer last meal–bedtime intervals were associated with lower dysmenorrhea (β = −0.131, *p* = 0.010; β = −0.111, *p* = 0.025, respectively (Figure 2B). The overall model was statistically significant for free days (*R*^2^ = 0.055, adjusted *R*^2^ = 0.028, *p* = 0.022), whereas no association was observed on workdays (*R*^2^ = 0.033, adjusted *R*^2^ = 0.007, *p* = 0.240).

For eating-related PMS symptoms, a longer wake–first meal interval on workdays was positively associated with higher eating-related PMS symptoms (β = 0.138, *p* = 0.008; Figure 2C). No associations were observed for the last meal–bedtime interval. The model was statistically significant for workdays (*R*^2^ = 0.047, adjusted *R*^2^ = 0.022, *p* = 0.046), whereas no association was observed for free days (*R*^2^ = 0.042, adjusted *R*^2^ = 0.015, *p* = 0.110).

For sleep-related PMS symptoms, no clear associations were observed between meal and sleep interval timing on either workdays or free days (Figure 2D). The models were not statistically significant for workdays (*R*^2^ = 0.029, adjusted *R*^2^ = 0.003, *p* = 0.361) or free days (*R*^2^ = 0.033, adjusted *R*^2^ = 0.006, *p* = 0.276).

Linear regression analyses were conducted to examine the associations of eating window duration and sleep duration with PMS symptoms and dysmenorrhea, after adjusting for age, BMI, smoking status, and alcohol consumption (Figure 2).

For the total PMS symptoms score, no associations were observed between eating window duration or sleep duration on either workdays or free days (Figure 2E). The overall models were not statistically significant for workdays (*R*^2^ = 0.025, adjusted *R*^2^ = −0.001, *p* = 0.475) or free days (*R*^2^ = 0.025, adjusted *R*^2^ ≈ 0, *p* = 0.481).

For dysmenorrhea, no association was observed between the eating window and sleep duration, and dysmenorrhea severity on workdays (*R*^2^ = 0.031, adjusted *R*^2^ = 0.005, *p* = 0.285). In contrast, on free days, longer eating window and longer sleep duration were associated with higher dysmenorrhea (β = 0.133, *p* = 0.007; β = 0.147, *p* = 0.004, respectively) (Figure 2F). The model was statistically significant on free days (*R*^2^ = 0.055, adjusted *R*^2^ = 0.030, *p* = 0.015).

For eating-related PMS symptoms, no associations were observed between the eating window and sleep duration, and eating-related PMS symptoms on either workdays or free days (Figure 2G). The models were not statistically significant for the workdays (*R*^2^ = 0.030, adjusted *R*^2^ = 0.004, *p* = 0.306) or free days (*R*^2^ = 0.030, adjusted *R*^2^ = 0.004, *p* = 0.315).

For sleep-related PMS symptoms, no associations were observed between eating window, sleep duration, and sleep-related PMS symptoms on workdays or free days (Figure 2H). The models were not statistically significant for workdays (*R*^2^ = 0.025, adjusted *R*^2^ = −0.001, *p* = 0.485) or free days (*R*^2^ = 0.037, adjusted *R*^2^ = 0.011, *p* = 0.160), although sleep duration on free days showed a marginal association with sleep symptoms (*p* = 0.073).

### 3.5 Associations of meal and snack Frequency with total PMS score, moderate-to-severe PMS status, eating and sleep-related PMS symptoms, dysmenorrhea, and chronotype (MSFsc)

**Figure 3:**
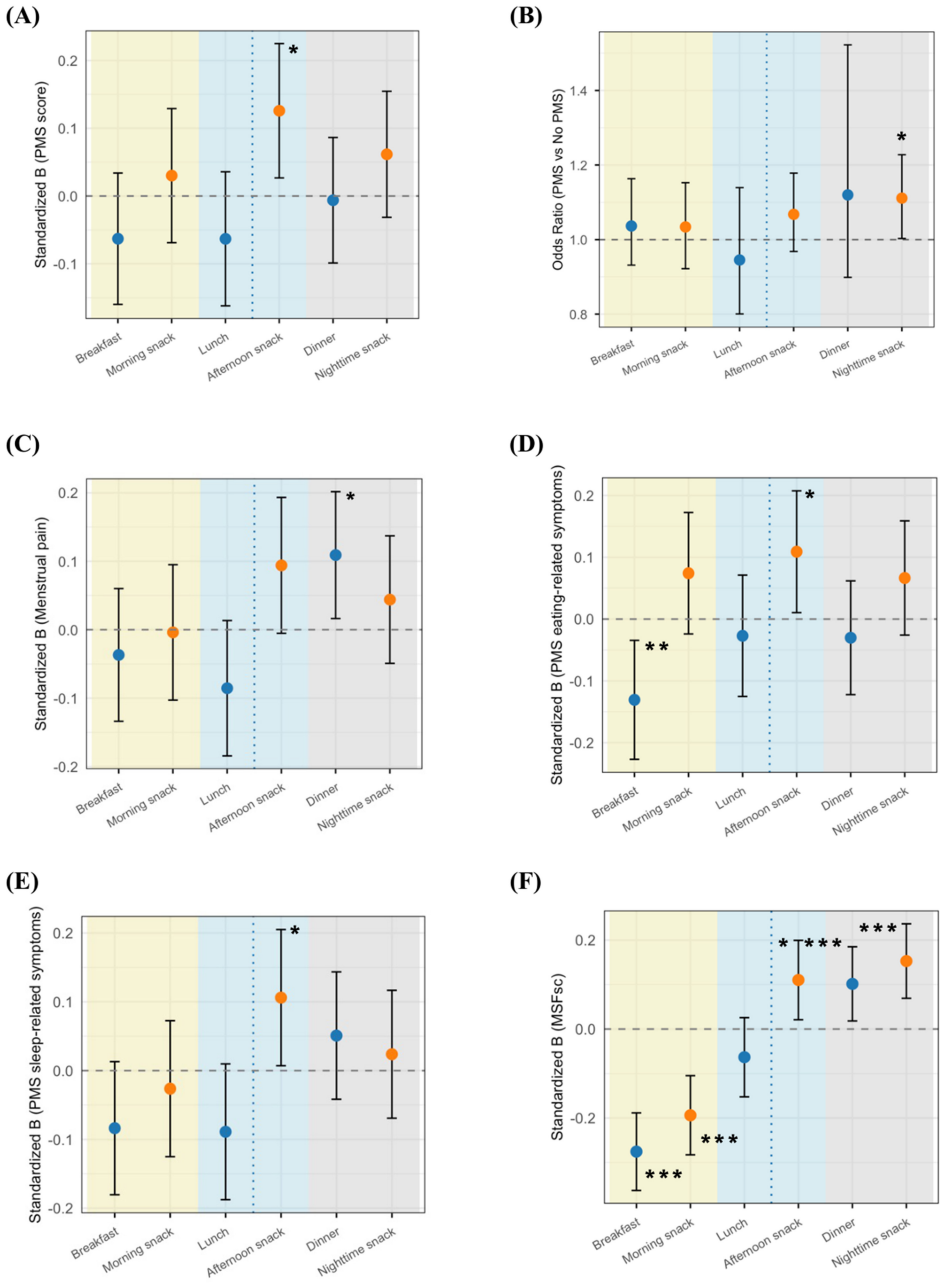
Associations of meal frequency and snack frequency with **(A)** total PMS score, **(B)** PMS status (moderate-to-severe PMS vs. No PMS), **(C)** menstrual pain (dysmenorrhea), **(D)** PMS eating-related symptoms (increased appetite, overeating, strong cravings), **(E)** PMS sleep-related symptoms (excessive sleep, difficulty waking up and poor sleep quality) and **(F)** MSFsc (Chronotype). **Panels A**, **C**, **D**, **E**, and **F** present standardized regression coefficients (β) from multivariable linear regression models, whereas **Panel B** presents odds ratios (ORs) from multivariable logistic regression models. All models were adjusted for age, BMI, smoking status, and alcohol consumption. Positive β values indicate higher symptom severity, whereas negative β values indicate lower symptom severity. OR > 1 indicates higher odds of moderate-to-severe PMS symptoms. The dashed horizontal line represents the null association (β = 0; OR =1 for **Panel B**). Statistical significance is denoted by *p < 0.05, **p < 0.01, ***p < 0.001.

In multivariate linear regression analyses adjusted for age, BMI, smoking status, and alcohol consumption, afternoon snack frequency was associated with a higher PMS symptoms score (β = 0.13, SE = 0.050, *p* = 0.013; Figure 3A). No associations were observed for breakfast, morning snack, lunch, dinner, or nighttime snack frequency (all *p* > 0.19). The overall model was statistically significant, but explained a modest proportion of the variance (*R*^2^ = 0.047; adjusted *R*^2^ = 0.026; *p* = 0.014).

In the multivariate logistic regression analyses, no associations remained after adjustment for multiple testing. Nighttime snack frequency was associated with higher odds of moderate-to-severe PMS symptoms in unadjusted analysis (OR = 1.11, 95% CI: 1.00–1.23, *p* = 0.040). Overall, effect estimates across meal and snack timing were small, and confidence intervals were close to unity.

For dysmenorrhea, dinner frequency was positively associated with dysmenorrhea severity (β = 0.11, SE = 0.047, *p* = 0.021; Figure 3C). Afternoon snack and lunch frequency showed borderline associations (*p* = 0.063 and *p* = 0.090, respectively). However, the overall model was not statistically significant (*p* = 0.207) and demonstrated limited explanatory power (adjusted *R*^2^ = 0.007), therefore this finding should be interpreted cautiously.

Afternoon snack frequency was positively associated with both eating-related (β = 0.11, *p* = 0.030; Figure 3D) and sleep-related PMS symptoms (β = 0.11, *p* = 0.036; Figure 3E). Breakfast frequency was inversely associated with eating-related PMS symptoms (β = −0.13, *p* = 0.008) and showed borderline inverse associations with sleep-related PMS symptoms. These models were statistically significant overall (eating-related: adjusted *R*^2^ = 0.039, *p* = 0.002; sleep-related: adjusted *R*^2^ = 0.021, *p* = 0.030), although the variance explained was only modest.

In contrast to PMS symptoms and dysmenorrhea, chronotype (MSFsc) showed the most consistent associations with meal and snack frequency (Figure 3F). Higher breakfast and morning snack frequency were associated with earlier chronotype (β = −0.275 and β = −0.194, respectively; both *p* < 0.001), whereas afternoon snack, dinner, and nighttime snack frequency were associated with later chronotype (β ranging from 0.102 to 0.153; all *p* ≤ 0.017). The overall model demonstrated moderate explanatory capacity (adjusted *R*^2^ = 0.187; *p* < 0.001).

## 4 Discussion

### 4.1 Main findings

In this cross-sectional study of Japanese women aged 19–39 years old, we examined meal and snack timing, sleep timing, chronotype, and social jetlag, and their association with PMS symptoms and dysmenorrhea. To the best of our knowledge, this is the first study to simultaneously investigate the meal and sleep timings across workdays and free days in relation to PMS symptoms and dysmenorrhea. Our main findings were: on workdays, 1) a later time of the first meal and 2) an earlier wake-up time were associated with higher eating-related PMS symptoms. In contrast, on free days, 3) an earlier time of the first meal, 4) a later time of the last meal, 5) a longer eating window, 6) a later wake-up time, and 7) a longer sleep duration were associated with greater dysmenorrhea severity. In addition, 8) higher breakfast frequency was associated with lower eating-related PMS symptoms. We also found that 9) more afternoon snacking was associated with a higher total PMS score, eating-related PMS and sleep-related PMS symptoms, whereas 10) more nighttime snacking was associated with higher odds of moderate-to-severe PMS symptoms.

### 4.2 Associations of meal timing, meal and snack frequency, and BMI with PMS symptoms and dysmenorrhea

In the present study, we observed significant differences in meal timing behavior between workdays and free days. Participants demonstrated a later first meal, a later last meal, and a shorter eating window on free days than on workdays (Table 1). Later time of first meal, particularly during socially imposed schedules, is associated with higher eating-related PMS symptoms, including higher appetite, overeating, or experiencing strong cravings (Figure 1). Lower breakfast frequency, a longer interval between wake-up time and first meal, and more afternoon and nighttime snacking were also associated with higher eating-related PMS symptoms (Figures 2 extbfand 3). Eating-related PMS symptoms, such as constantly feeling hungry, overeating, or experiencing strong cravings for specific foods, were the most prevalent moderate to severe symptoms reported in this cohort (Table 1; Supplementary File 1).

During workdays, participants with earlier wake times and later eating patterns may exhibit circadian misalignment arising from discrepancies between the socially imposed schedule and endogenous circadian preference (49). Early-morning food intake is an important synchronizing *zeitgeber* for peripheral clocks in metabolic tissues, including the liver, adipose tissue, skeletal muscle, and gastrointestinal tract, coordinated by the central circadian clock located in the suprachiasmatic nucleus (50, 51). Later or irregular early-day eating has been associated with reduced synchronization between central and peripheral clocks, potentially impairing glucose metabolism and appetite regulation (52). Circadian misalignment has been associated with altered leptin-ghrelin balance and impaired insulin sensitivity (53), which may provide one possible explanation for the observed association between later first meal timing and higher eating-related PMS symptoms in this study.

These metabolic alterations may interact with reproductive hormonal fluctuations during the luteal phase and have been proposed as potential mechanisms underlying mood changes, appetite dysregulation, and increased snacking (54). In the present cohort, 72.5% of participants reported an increase in appetite during the late luteal phase, suggesting pronounced behavioral changes during this period (Supplemental File 1). However, the menstrual cycle phase was not assessed at the time of symptom reporting; therefore, residual confounding due to variation in menstrual cycle phase cannot be excluded. Participants experiencing pronounced late luteal-phase symptoms at the time of assessment may have been more likely to report higher snacking behavior, which could have contributed to the observed associations between snacking behavior and PMS symptoms. Previous studies have reported increased energy intake during the late luteal phase, characterized by increased consumption of sugary foods and snacks and greater overall macronutrient intake (55, 56). We observed a similar pattern, with differential associations between snack timing and PMS symptom severity and moderate-to-severe PMS status according to the timing of snack consumption (Figure 3). Morning snacking was not associated with PMS symptom severity or moderate-to-severe PMS status (Figures 3A and 3B). Afternoon snacking was associated with higher PMS symptom severity, whereas nighttime snacking was associated with higher odds of moderate-to-severe PMS status (Figures 3A and 3B). Frequent nighttime snacking has been associated with circadian misalignment by extending the eating window into the biological night (53), a period characterized by reduced insulin sensitivity and metabolic efficiency (57, 58). While causal inference cannot be drawn, these associations are consistent with emerging evidence that late-day energy intake is associated with circadian misalignment (51, 52). The circadian system plays a central role in hormone secretion, inflammatory responses, pain perception, and mood regulation (18–20). Therefore, circadian misalignment associated with nighttime eating may represent one potential mechanism underlying the observed association with PMS symptoms. However, the present analysis did not account for the nutritional composition of meals and snacks at each timing, which should be addressed in future studies.

Previous studies have demonstrated a positive association between PMS and adiposity (59). BMI was not related to PMS severity in our normal-weight cohort. Recent randomized controlled trials (RCTs) on alternate-day fasting and caloric restriction have reported reductions in PMS symptoms and improvements in menstrual-related hormonal outcomes among women with obesity (57, 60–62). In addition, a systematic review reported that time-restricted eating (TRE) may influence reproductive hormones, with evidence suggesting reduced androgen-related markers and increased sex hormone-binding globulin, but no consistent effects on estrogen and gonadotropins in premenopausal women with obesity (63). Given that our cohort consisted of normal-weight women, the absence of an association with BMI suggests that the observed associations between behavioral timings and menstrual symptoms were not fully explained by adiposity in this cohort. Future RCTs should investigate whether chrono-nutritional interventions, such as optimizing meal timing and reducing late-night eating, can alleviate PMS symptoms in normal-weight populations.

Although dietary quality and skipping breakfast have been linked to dysmenorrhea, the role of food intake timing has received relatively limited attention (4, 33, 64–66). Notably, while previous work has primarily focused on what is eaten, the present study uniquely addresses when food is consumed, providing a novel chronobiological perspective on dysmenorrhea. In the present study, dysmenorrhea was associated with a later last meal and a longer eating window, particularly on free days (Figures 1 and 2). One possible pathway involves melatonin, a central circadian hormone secreted in response to the light–dark cycle. Beyond its role in sleep regulation, melatonin has been reported to exert analgesic and anti-inflammatory effects that may influence pain regulation (67, 68). Because melatonin secretion follows a robust circadian rhythm, shifts in meal timing have been associated with alterations in normal nocturnal melatonin secretion patterns (52). However, given the cross-sectional design of the present study and the mixed clinical evidence regarding the analgesic and antinociceptive effects of melatonin (68, 69), these mechanisms remain unclear.

In addition, later meal timing and shorter fasting windows have been associated with impaired insulin sensitivity, glucose homeostasis, and systemic inflammation, which may be relevant to the pathophysiology of dysmenorrhea (48). Breakfast skipping has been shown to increase postprandial glucose excursions and impair glycemic control (53). Such metabolic dysregulation may contribute to systemic low-grade inflammation, which has been associated with higher PMS severity in women (70). Together, these pathways may provide plausible explanations for the observed associations between meal timing and dysmenorrhea.

### 4.3 Associations of sleep timing, chronotype, and social jetlag with PMS and dysmenorrhea

Although the role of sleep timing remains unexplored, numerous studies have reported an association between sleep quality and menstrual symptoms. A previous study reported daytime sleepiness has been reported to increase during the luteal phase (31), and both sleep quality and duration have been associated with the severity of perimenstrual symptoms (31, 71). A recent study demonstrates that sleep irregularity, shorter sleep duration, and poorer sleep quality were significantly associated with both PMS and dysmenorrhea (72). A recent systematic review reported altered circadian rhythm among women with PMS, including lower melatonin levels, elevated body temperature, and poorer sleep quality (73).

We observed significant differences in sleep timing behavior between workdays and free days in this population. Participants wake up and eat their first meal earlier on workdays (Table 2). Earlier wake-up times and longer intervals between wake-up times on and time of first meal on workdays were associated with eating-related PMS symptoms, such as overeating, strong cravings, and higher appetite (Figures 1 extbfand 2). Earlier wake-up times and later food intake in the morning during workdays may reflect behavioral patterns associated with circadian misalignment, which has been linked to altered metabolic and hormonal regulation (52, 53). Such associations may be particularly relevant during the luteal phase due to the reproductive hormonal fluctuations (74), contributing to greater eating-related PMS symptoms on days with socially imposed schedules.

In the present study, chronotype was not significantly associated with PMS symptoms or dysmenorrhea. Similarly, no significant association were observed between social jetlag and PMS symptoms or dysmenorrhea. These findings differ from previous studies reporting that evening chronotype has been linked to greater PMS severity and increased dysmenorrhea risk, and a morning chronotype is associated with less severe menstrual symptoms (36, 39, 40, 75–77). Despite the absence of a direct association between chronotype and menstrual symptoms, we observed that later wake-up times and longer sleep durations on free days, was associated with dysmenorrhea (Figures 1 and 2). Sleep disruption and greater variability in sleep-wake patterns in evening chronotype may explain the higher risk of dysmenorrhea through impaired hormonal regulation (40). Melatonin has been shown to be associated with pain regulation (67, 68). Nevertheless, these findings remain speculative in the context of present findings because chronotype and social jetlag were not directly associated with PMS symptoms or dysmenorrhea. Reverse causality or a bidirectional relationship may also explain these findings, as dysmenorrhea could influence sleep behavior. For example, menstrual pain may disrupt nighttime sleep, leading to compensatory longer sleep duration during unconstrained free days (26, 78).

The lack of association between social jetlag and menstrual symptoms may be partly explained by the relatively low levels of social jetlag observed in our sample, with approximately 60% of participants exhibiting < 1 h of social jetlag. A previous study among university students reported that >1 h of social jetlag was significantly associated with more severe menstrual symptoms independent of sleep duration and chronotype (37). However, another study in nursing students, in which approximately 70% of participants reported > 1 h of social jetlag, reported not significant association with PMS and menstrual symptoms, indicating inconsistent evidence (79). Our findings suggest that sleep timing behavior, particularly on free days, may be more closely related to dysmenorrhea than chronotype or social jetlag. Future longitudinal studies incorporating chronotype and social jetlag on menstrual symptoms are needed to clarify these relationships.

Overall, the highlights of this study are the differential associations observed between meal and sleep timings in relation to PMS symptoms and dysmenorrhea across workdays and free days. These findings suggest that eating-related PMS symptoms may be more closely associated with behaviors shaped by socially imposed schedules, whereas dysmenorrhea may be more closely associated with behavioral patterns that reflect endogenous circadian preference. On workdays, earlier wake-up times combined with a later first meal and a longer interval between waking and eating were associated with higher eating-related PMS symptoms. One possible explanation is that these behavioral patterns may be linked to misalignment between the social clock and the biological clock (49), which has been associated with disrupted central–peripheral clock coordination (50), impaired glucose metabolism and insulin sensitivity (52), and altered appetite regulation (54). Such mechanisms may be particularly relevant during the late luteal phase, when reproductive hormone fluctuations occur (74). In contrast, on free days, which may closely reflect endogenous circadian preference, behavioral patterns such as later wake-up times, prolonged eating windows, and shorter fasting windows were associated with greater dysmenorrhea severity. The patterns have been previously linked to eveningness-related behavior (36, 40), metabolic dysregulation (48), systemic low-grade inflammation (70), altered melatonin rhythms (67, 68), and compensatory sleep behaviors (11, 26, 78), providing possible pathways that may explain the observed association with greater dysmenorrhea severity. Notably, these relationships may be bidirectional, as menstrual symptoms themselves may also influence meal timing and sleep behaviors (26). In addition, the modest explanatory power of the regression models indicates that meal timing and sleep timing likely account for only a small proportion of variability in PMS and dysmenorrhea. Although causality cannot be inferred from the cross-sectional design, these findings support the growing literature suggesting that meal timing and sleep timing are associated with menstrual health outcomes, warranting further investigation in longitudinal and interventional studies.

### 4.4 Strengths and limitations

This study has several strengths. A key strength of this study is the assessment of meal and sleep timings across workdays and free days. By examining behaviors reflecting socially imposed schedule (workdays) and endogenous circadian preference (free days) behaviors, this study provides insights into how they differentially associate with menstrual symptoms. Second, the inclusion of snack timing, in addition to main meals (breakfast, lunch, dinner) timing, enabled more comprehensive patterns of temporal daily eating patterns.

However, this study has several limitations. First, this study employed a cross-sectional study design; therefore, causal relationships and reverse causality could not be established. Longitudinal studies or intervention trials are needed to clarify causal relationships between meal and sleep timing and menstrual health outcomes. Second, demographic characteristics, as well as meal and sleep timing variables, were self-reported, introducing a potential recall bias and measurement error. Participants reported their habitual patterns of their eating and sleep behaviors, rather than objectively measured behaviors. Third, the menstrual cycle phase was not assessed or adjusted for. Although PMS symptoms and dysmenorrhea were reported with reference to specific phases of the menstrual cycle, meal and sleep timing were assessed at the time of the questionnaire completion and may vary across the menstrual cycle. Therefore, residual confounding due to cycle-related variation in eating and sleep behaviors cannot be excluded. Future studies should account for menstrual cycle phase and prospectively assess behavioral patterns across the menstrual cycle. Fourth, although the analyses were adjusted for age, alcohol intake, BMI, and smoking status, residual confounding from other factors cannot be excluded. In particular, dietary intake, sleep quality, physical activity level, caffeine intake, use of oral contraceptives or hormone replacement therapy, and stress or mental health status were not included in the regression analysis and may have influenced the observed associations. Furthermore, information regarding the menstrual regularity, cycle length, gynecological disorders (e.g., endometriosis and PCOS), and hormonal medication use was not available. Although analgesic use during menstruation was collected descriptively, it was not included in the regression analyses. Fifth, the study population may have been biased toward more health-conscious individuals who were willing to record their behaviors using a mobile application. Sixth, the regression models explained only a modest proportion of the variability in PMS symptoms and dysmenorrhea (*R*^2^ = 0.013 to 0.08), suggesting that these outcomes are influenced by other factors not assessed in this study, therefore the findings should be interpreted cautiously. Seventh, although dysmenorrhea was assessed using a single-item 4-point ordinal scale widely used from previous studies assessing dysmenorrhea (43–46), this scale may provide limited granularity compared with the psychometric pain measures like the visual analog scale (VAS). Eighth, formal multiple-testing adjustment methods were not applied because strict correction may be overly conservative given the correlated nature of the exposure and outcomes. Therefore, findings should be interpreted as exploratory and hypothesis-generating, with emphasis on effect sizes, confidence intervals, and overall patterns rather than *p*-values. Finally, the study was conducted in Japanese women, which may limit the generalizability of the findings to other populations.

## 5 Conclusion

Meal timing and sleep timing were differentially associated with PMS symptoms and dysmenorrhea across workdays and free days. Workday behaviors were primarily associated with eating-related PMS symptoms, whereas free-day behaviors were primarily associated with dysmenorrhea severity. These findings suggest that menstrual symptoms may be differentially associated with behaviors shaped by socially imposed schedules and endogenous circadian preferences. Future longitudinal and interventional studies are needed to clarify causal relationships between meal timing, sleep timing, and menstrual health outcomes.

## Data Availability

The original contributions presented in the study are included in the article/Supplementary material, further inquiries can be directed to the corresponding author.
