# Supplementary material for: Associations of meal timing and sleep timing with premenstrual syndrome and dysmenorrhea in Japanese women: a cross-sectional study
Source: Front Nutr. 2026 Jul 24;13:1875559. doi: 10.3389/fnut.2026.1875559 (PMC13449302; doi:10.3389/fnut.2026.1875559)
Supplement: Supplementary file 1 [file Supplementary_File_1.docx]

|  | Domains | Never | Mild | Moderate | Severe |
| --- | --- | --- | --- | --- | --- |
| Premenstrual symptoms 1–2 weeks before menstruation | | | | | |
| 1 | Felt sad, depressed, gloomy, hopeless, felt worthless, or felt excessive guilt. | 165 (35.1) | 150 (31.9) | 114 (24.3) | 41 (8.7) |
| 2 | Felt anxious, tense, excited, or irritable. | 100 (21.3) | 167 (35.5) | 141 (30) | 62 (13.2) |
| 3 | Suddenly felt sad or became tearful; felt overly sensitive to rejection or emotionally vulnerable. | 191 (40.6) | 131 (27.9) | 94 (20) | 54 (11.5) |
| 4 | Felt angry or became more easily angered. | 105 (22.3) | 167 (35.5) | 126 (26.8) | 72 (15.3) |
| 5 | Felt overwhelmed, unable to cope well, or felt a loss of self-control. | 266 (56.6%) | 112 (23.8) | 63 (13.4) | 29 (6.2) |
| 6 | Lost interest in usual activities (friends, hobbies, school. | 241 (51.3) | 137 (29.1) | 67 (14.3) | 25 (5.3) |
| 7 | Had difficulty concentrating. | 202 (43) | 156 (33.2) | 87 (18.5) | 25 (5.3) |
| 8 | Felt lethargic, bored, or fatigued. | 138 (29.4) | 147 (31.3) | 128 (27.2) | 57 (12.1) |
| 9 | Slept too much or had difficulty waking up in the morning; or had trouble falling asleep or woke up during the night. | 266 (56.6) | 112 (23.8) | 63 (13.4) | 29 (6.2) |
| 10 | Felt constantly hungry and overate, or experienced strong cravings for specific foods. | 111 (23.6) | 132 (28.1) | 132 (28.1) | 95 (20.2) |
| 11 | Experienced breast tenderness or pain; headaches, joint pain, or muscle pain; noticed weight gain or body swelling. | 94 (20) | 159 (33.8) | 151 (32.1) | 66 (14) |
| Types of difficulties due to having premenstrual symptoms | | | | | |
| 12 | Reduced productivity in daily life at work, school, or home (work, study, or housework did not go well). | 145 (30.9) | 196 (41.7) | 107 (22.8) | 22 (4.7) |
| 13 | Stopped participating in hobbies, club activities, or social activities, or participated less frequently. | 265 (56.4) | 127 (27) | 57 (12.1) | 21 (4.5) |
| 14 | Experienced difficulties in relationships with others. | 304 (64.7) | 110 (23.4) | 40 (8.5) | 16 (3.4) |

Data are presented as a number (percentage)

**Supplementary Table 1.** Frequency of each domain of Premenstrual Syndrome (PMS) symptoms

|  | Variables | Frequency (%) | |
| --- | --- | --- | --- |
| Menstrual pain (dysmenorrhea) | | |  |
| 1 | None | 136 (29) | |
| 2 | Slight interference with work or academic activities | 245 (52.2) | |
| 3 | Interferes with work or academic activities to the extent that you want to lie down and rest temporarily | 80 (17.1) | |
| 4 | Unable to work or attend school | 8 (1.7) | |
| Use of painkillers during menstruation | | |  |
| 1 | Do not use | 166 (35.4) | |
| 2 | Rarely (about 1–2 times per menstrual period) | 195 (41.6) | |
| 3 | Sometimes (about 3–5 times per menstrual period) | 76 (16.2) | |
| 4 | Frequently (6 or more times per menstrual period) | 32 (6.8) | |
| Appetite before menstruation | | |  |
| 1 | My appetite increases | 340 (72.5) | |
| 2 | My appetite is the same as usual | 100 (21.3) | |
| 3 | My appetite decreases | 6 (1.3) | |
| Physical and health condition† | | | |
| 1 | My weight sometimes increases by 2 kg or more before menstruation. | 115 (24.5) | |
| 2 | I have been told by a doctor that I have anemia. | 129 (27.4) | |
| 3 | I am sensitive to cold / have cold intolerance. | 217 (46.2) | |
| 4 | My weight has increased or decreased by 3 kg or more within the past year. | 190 (40.4) | |
| 5 | None of the above apply. | 88 (18.7) | |

Data are presented as number (percentage). †Participants could select more than one physical or health condition; therefore, percentages do not sum to 100%.

**Supplementary Table 2.** Frequency of menstrual pain (dysmenorrhea), painkiller use during menstruation, appetite before menstruation, and physical health conditions.
